# Role of tissue transglutaminase 2 in the acquisition of a mesenchymal-like phenotype in highly invasive A431 tumor cells

**DOI:** 10.1186/1476-4598-10-87

**Published:** 2011-07-21

**Authors:** Chun-Yu Lin, Pei-Hsun Tsai, Chithan C Kandaswami, Geen-Dong Chang, Chia-Hsiung Cheng, Chang-Jen Huang, Ping-Ping Lee, Jiuan-Jiuan Hwang, Ming-Ting Lee

**Affiliations:** 1 Institute of Biochemical Sciences, National Taiwan University, Taipei, Taiwan; 2 Castle Hills Health, 2267 Sir Amant Drive, Lewisville, TX 75056, USA; 3 Institute of Biological Chemistry, Academia Sinica, Taipei, Taiwan; 4 Institute of Physiology, National Yang-Ming University, Taipei, Taiwan

**Keywords:** epithelial-mesenchymal transition, tissue transglutaminase, matrix metalloproteinase, PI3K/Akt, NF-κB, Snail, migration

## Abstract

**Background:**

Cancer progression is closely linked to the epithelial-mesenchymal transition (EMT) process. Studies have shown that there is increased expression of tissue tranglutaminase (TG2) in advanced invasive cancer cells. TG2 catalyzes the covalent cross-linking of proteins, exhibits G protein activity, and has been implicated in the modulation of cell adhesion, migration, invasion and cancer metastasis. This study explores the molecular mechanisms associated with TG2's involvement in the acquisition of the mesenchymal phenotype using the highly invasive A431-III subline and its parental A431-P cells.

**Results:**

The A431-III tumor subline displays increased expression of TG2. This is accompanied by enhanced expression of the mesenchymal phenotype, and this expression is reversed by knockdown of endogenous TG2. Consistent with this, overexpression of TG2 in A431-P cells advanced the EMT process. Furthermore, TG2 induced the PI3K/Akt activation and GSK3β inactivation in A431 tumor cells and this increased Snail and MMP-9 expression resulting in higher cell motility. TG2 also upregulated NF-κB activity, which also enhanced Snail and MMP-9 expression resulting in greater cell motility; interestingly, this was associated with the formation of a TG2/NF-κB complex. TG2 facilitated acquisition of a mesenchymal phenotype, which was reversed by inhibitors of PI3K, GSK3 and NF-κB.

**Conclusions:**

This study reveals that TG2 acts, at least in part, through activation of the PI3K/Akt and NF-κB signaling systems, which then induce the key mediators Snail and MMP-9 that facilitate the attainment of a mesenchymal phenotype. These findings support the possibility that TG2 is a promising target for cancer therapy.

## Background

The epithelial-mesenchymal transition (EMT), first recognized as a hallmark of embryogenesis in the early 1980, is a crucial morphogenic process during embryonic development [1,2]. During the EMT, the non-motile polarized epithelial cells that originally display many cell-cell junctions lose contact with each other and gradually convert into individual, non-polarized, motile, and invasive mesenchymal cells [3]. There is growing acceptance that the detachment of single carcinomatous cells and their migration into the stroma replicates the developmental EMT process [4-6]. The EMT is a vibrant, dynamic and transient process, and therefore the process manifests as epithelial cell plasticity during tumor progression. A striking characteristic of the EMT is the loss of E-cadherin expression, an important caretaker of the epithelial phenotype [1]. Several transcription factors have been implicated in the transcriptional repression of E-cadherin, including the zinc finger proteins of the Snail/Slug family, Twist, δEF1/ZEB1, SIP1, and the basic helix-loop-helix factor E12/E47 [4,7]. These repressors also act as molecular triggers of the EMT program by repressing a subset of common genes that encode cadherins, claudins, cytokines, integrins, mucins, plakophilin, occludin, and zonula occludens proteins, thereby promoting EMT. All of these transcription factors have been duly recognized as playing a critical role in cell survival, differentiation, and metastasis.

Tissue transglutaminase (TG2/tTG), a member of the transglutaminase family, is a calcium-dependent enzyme that catalyzes the covalent cross-linking of proteins. This multifunctional protein is expressed ubiquitously and abundantly, and has been implicated in a variety of cellular processes, such as cell differentiation, death, inflammation, migration, and wound healing [8-12]. Patients suffering from cancers may become refractory to anticancer agents (drug resistance) following chemotherapy or undergo cancer cell metastasis. Researchers have noticed that cancer cells exhibiting resistance to anticancer drugs together with those that are isolated from metastatic sites have relatively higher TG2 expression levels [13-16]. Additionally, down-regulation of TG2 by gene-specific siRNA, antisense RNA or ribozyme approaches reverses drug-resistance in breast, pancreatic, lung, and ovarian carcinoma cells [17-22]. Recently, Shao and coworkers documented that TG2 modulated the EMT and contributed to increased ovarian cancer cell invasiveness and tumor metastasis [23]. They showed that TG2 induced Zeb1 by activating the NF-κB complex. The effects of TG2 on ovarian cancer cell phenotype and invasiveness translated into increased metastasis and tumor formation *in vivo*, as assessed in an orthotopic ovarian xenograft model. Kumar and coworkers also have shown that aberrant expression of TG2 is sufficient to induce the EMT in epithelial cells, and they also established a strong link between TG2 expression and progression of metastatic breast disease [24]. The nature of TG2 involvement in the EMT has not been well elucidated. Nevertheless, the above studies provide evidence implying that TG2 promotes EMT and enhances tumor metastasis by activating oncogenic signaling.

We have isolated a highly invasive tumor cell subline (A431-III) from parental A431 tumor cells (A431-P) using a Boyden chamber system with matrigel-coated membrane support. These A431-III cells secrete a higher level of MMP-9 and exhibit greater adhesion, spreading, migration, and invasive capability compared to A431-P cells [25]. Based on the above, A431-P cells and A431-III subline should be able to serve as a model system that will help to delineate the mechanisms involved in the EMT. We observed that MMP-9-induced acquisition of an invasive phenotype in A431-III cells was associated with marked and decisive increases in the levels of fibronectin and TG2 [26]. In addition, our most recent study produced an interesting finding whereby MMP-9 and Snail form a mutual regulatory loop, and work cooperatively within the EMT induction process [27]. Since highly invasive A431-III cells display enhanced expression of TG2 [26], and TG2 expression modulates the EMT [23,24], we were prompted to explore the role of TG2 in the induction of the EMT in A431-P and A431-III cells.

In this study we have demonstrated that TG2 participates in the acquisition of the mesenchymal phenotype in A431-P and A431-III cells. We propose that TG2, acting via activation of NF-κB and PI3K/Akt-GSK3β signaling, enhances the expression of Snail, and that this leads to the acquisition of mesenchymal phenotype in A431-III cells. This in turn promotes MMP-9 activity, which increases cancer cell motility and metastatic potential. This and other studies support the contention that TG2 is a promising therapeutic target for studies that explore reversing drug resistance and inhibiting the metastatic potential of tumor cells.

## Methods

### Materials

The A431 tumor cell line was obtained from the American Type Culture Collection (ATCC; Manassas, VA). The epidermoid carcinoma cell line A-431 was originally derived from a cervical solid tumor of an 85-year-old female [28]. TG2 siRNA, and non-specific siRNA were purchased from Invitrogen (Carlsbad, CA). Anti-TG2 was purchase from Thermo Scientific (Fremont, CA). Anti-Snail was obtained from Abcam (Cambridge, MA) and anti-N-cadherin was purchased from Abgent (San Diego, CA). Anti-fibronectin and anti-β-actin were purchased from Sigma (St. Louis, MO). Anti-vimentin (V9) and anti-IκBα were obtained from Santa Cruz (Santa Cruz, CA). Anti-p-Akt(Ser473), anti-p-GSK3β(Ser9), anti-Lamin A, and anti-cyclin D1 were obtained from GeneTex (Irvine, CA). Anti-Akt was obtained from Cell Signaling (Boston, MA). Anti-NF-κB and anti-GSK3βwere obtained from BD Transduction (Franklin Lakes, NJ). All PCR forward and reverse primers were purchased from Purigo Biotech (Taipei, Taiwan).

### Preparation of cell lysates and nuclear extracts

The cells were lysed in gold lysis buffer, containing 20 mM Tris-HCl (pH 7.9), 1 mM EGTA, 0.8% NaCl, 0.1 mM β-glycerylphosphate, 1 mM sodium pyrophosphate, 10 mM NaF, 1 mM Na_4_P_2_O_7_, 1 mM Na_3_VO_4_, 10% glycerol, 1% Triton X-100, 1 mM PMSF, 10 μg/ml aprotinin, and 10 μg/ml leupeptin. Insoluble material was separated by centrifugation at 14,000 × *g *for 20 min at 4°C. Protein concentrations were determined using the method of Bradford [29].

The nuclear fraction extraction procedure was performed as described by Schreiber et al. [30]. Briefly, the cell pellets were resuspended in 400 μL of buffer A, containing 10 mM HEPES (pH 7.9), 10 mM KCl, 0.1 mM EDTA, 0.1 mM EGTA, 1 mM DTT, PMSF 1 mM. The cells were incubated on ice 15 min and then 25 μL of 10% NP-40 was added. The cells were centrifuged at 500 × *g *for 5 min. The supernatant, which contains the cytoplasmic fraction, was then collected. The nuclear pellet was resuspended in 50 μL of cold buffer B, containing 20 mM HEPES (pH 7.9), 0.4 M NaCl, 1 mM EDTA, 1 mM EGTA, 1 mM DTT, 1 mM PMSF. The vials then rocked vigorously on a shaking platform for 15 min, which was followed by centrifugation at 500 × *g *for 5 min. The supernatant nuclear fraction was then collected.

### Western blotting

Protein samples were separated on 10% SDS-polyacrylamide gels. The membrane blots were blocked in PBS containing 5% BSA for 1 h at room temperature, and incubated with primary antibody overnight at 4°C. After washing with TBST containing 20 mM Tris-HCl (pH 7.6), 0.8% (w/v) NaCl, and 0.25% Tween-20, the blots were incubated with secondary antibody conjugated with horseradish peroxidase. The immunoreactive bands were detected with ECL reagents (Millipore, Billarica, MA) and exposed using Fujifilm (Tokyo, Japan). The relative quantification of the ECL signals on the X-ray film was carried out by Image J software (NIH, Bethesda, MD).

### Reverse transcriptase-polymerase chain reaction (RT-PCR)

Total RNA was isolated using a PureLink RNA Mini Kit (Invitrogen, Carlsbad, CA), and reverse transcribed using a MMLV High Performance Reverse Transcriptase kit (Epicentre, Madison, WI). PCR amplication was performed over 20-40 cycles that consisted of denaturation at 94°C for 30s, annealing at 55°C to 60°C for 30s, and extension at 72°C for 30s-60s. Forward and reverse primers for the gene cDNA amplification are listed in the Table 1. The PCR products were separated on 1% agarose gels, stained with SYBR safe DNA stain (Invitrogen), and visualized under UV light.

**Table 1:** The forward and reverse primers of genes

Gene Name	Forward and Reverse primers	Amplified size(bps)
MMP-9	F 5'-TCTTCCCTGGAGACCTGAGAAC-3'	428
	R 5'-GACACCAAACTGGATGACGATG-3'	
Snail	F 5'-GCTCCTTCGTCCTTCTCCTCTA-3'	390
	R 5'-GGCACTGGTACTTCTTGACA-3'	
TG2	F 5'-GGAGGATATCACCCACACCTACA-3'	361
	R 5'-CGTAAGGCAGTCACGGTATTTC-3'	
GAPDH	F 5'-CCATCACTGCCACCCAGAAGA-3'	439
	R 5'-TCCACCACCCTGTTGCTGTA-3'	

### Gene construction and transfection

The full length cDNA encoding TG2 was isolated from human cervical epithelial cancer cell A431-III cDNA by RT-PCR using the specific primers, hTG2-F, 5'-AGGAGCCACCGCCCCCGCCCGACCATGGCC-3' and hTG2-R, 5'-CAGCAGGCTGGGAGCAGGGGTCCCTTAGGC-3'. The full length of TG2 was then cloned into the pGEMT-Easy vector (Promega, San Luis Obispo, CA) and identified by DNA sequencing. The coding region of TG2 was removed from the pGEMT-Easy vector using the restriction enzymes EcoRI and XhoI, and then subcloned into the EcoRI and XhoI sites of the pcDNA3.1 vector. Ligation of the restriction enzyme digested TG2 and pcDNA3.1 vector generated pcDNA3-TG2.

A431-P cells were seeded into 6-cm cultured dishes and then transfected with 4 μg of pcDNA3-TG2 using the Xfect transfection reagent (Clontech, Mountain View, CA) following the manufacturer's instructions. Expression of TG2 was screening by Western blotting and RT-PCR.

### Transfection of small interfering RNA (siRNA)

TG2 siRNA and non-specific siRNA were dissolved in RNase-free water provided by the manufacturer to a stock concentration of 20 μM. A431-P and A431-III cells were plated into 60 mm culture dishes and then transfected with 40 nM of siRNA using lipofectamine 2000 transfection reagent (Invitrogen, Carlsbad, CA) following the manufacturer's instructions. All assays were performed 48 h after transfection.

### NF-κB reporter luciferase assay

A431-P and A4331-III cells were seeded into 6-well plates. The cells were transfected with 2.5 μg of pNF-κB-Luc (Panomics, Dumbarton Circle Fremont, CA) or empty control vector using Xfect transfection reagent (Clontech), following the manufacturer's instructions. To detect the luciferase activity, the cells were lysed in luciferase cell-culture lysis reagent (Promega) and 50 μL of cell lysate was then mixed with 50 μL of luciferase assay substrate. The relative light units produced by each sample were detected by 1420 Luminescence Counter (Perkin Elmer, Waltham, MA). The sample data were normalized against the empty vector control and the protein concentrations.

### Gelatin zymography

Samples of conditioned media were subjected to electrophoresis on 8% SDS-polyacrylamide gels copolymerized with 0.1% gelatin. The volume of each medium sample analyzed was normalized according to the cell number. After electrophoresis, the gels were washed for 60 min in 2.5% Triton X-100, and incubated in reaction buffer (50 mM Tris-HCl, pH 8.0, containing 5 mM CaCl_2_, and 0.02% NaN_3_) at 37°C for 24 h. The gels were then stained with Coomassie Blue R-250 in 10% acetic acid/20% ethanol for 1 h, followed by destaining in the same solution without dye. A clear zone on the gel indicated the presence of gelatinase activity, which was then quantified by densitometry.

### Immunofluorescence staining

A431-P and III cells were plated into 6-well plates containing glass coverslips without a fibronectin coating. Following treatment with TG siRNA and non-specific siRNA, or following transfection with the TG2 expression vector, the cells were fixed with 4% paraformaldehyde. Cells were permeabilized with 0.1% Triton X-100 in PBS for 10 min. The permeabilized cells were then incubated with 3% BSA in PBS to block non-specific binding for 1 h at room temperature. After thorough rinsing with PBS, the cells were incubated with mouse monoclonal anti-vimentin and rabbit polyclonal anti-fibronectin antibodies at 4°C overnight. Next the cells were incubated with fluorescently labeled secondary antibodies for 1 h at room temperature in the dark. After rinsing with PBS, the cells were then stained with DAPI in PBS for 5 min at room temperature. The coverslips were then mounted using mounting medium on microslides and visualized by confocal microscopy.

### *In vitro *wound-healing migration assay

Both A431 and A431-III cells transfected with either TG2 siRNA or the full length TG2 expression vector were plated onto six-well culture plates in RPMI-1640 containing 10% FBS. After 24 h, the cell monolayers were wounded by manually scratching it with a pipette tip; this was followed by washing with PBS. The monolayers were then incubated at 37°C for 24 h. The monolayers were photographed at 0 h and 24 h after wounding using phase contrast microscopy and an Olympus IX70 camera. The experiments were performed in triplicate for each treatment group.

### Statistical analysis

The quantitative data derived from three to six independent experiments are expressed as means (± SEM). Unpaired Student's *t*-tests were used to analyze between group differences that is repeated and *p *< 0.05 was considered statistically significant.

## Results

Previously, we have demonstrated that TG2 and fibronectin are both upregulated in the highly invasive A431-III subline compared with the parental A431-P cells, and that knockdown of TG2 decreased integrin's association with fibronectin as well as reducing the level of MMP-9 and MMP-1; these events were accompanied by a reduction the A431-III cells' capability of undergoing adhesion, migration and invasion [26]. This prompted us to further explore the potential role of TG2 in the modulation of the EMT as well as the associated mechanisms using the A431-P and A431-III system that had been established in our laboratory.

### TG2 modulation of various EMT markers in A431-P and A431-III cells

**Figure 1:**
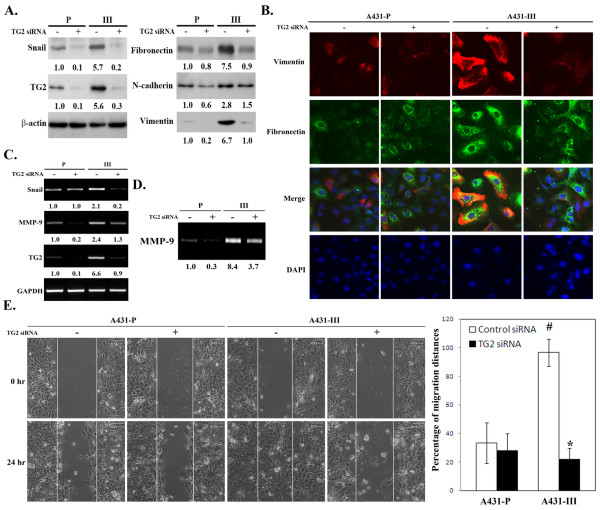
**Effect of TG2 knockdown on mesenchymal markers in A431-P and A431-III cells**. (A) The cells were treated with 40 nM of TG2-specific siRNA or control siRNA. At 48 h post-transfection, cell lysates were prepared and subjected to immunoblotting analysis for TG2, Snail fibronectin, N-cadherin, vimentin and β-actin served as internal controls. (B) The cells were plated onto non-fibronectin-coated cover slips in six-well plate for 24 h. The cells were treated with 40 nM of TG2 siRNA or control siRNA, and then immuno-stained for fibronectin (green) and vimentin (red) with the nuclei stained with DAPI (blue). The fluorescence images were visualized using confocal microscopy. (C) Total RNA was extracted at 48 h after siRNA transfection and analyzed for TG2, Snail and MMP-9 by RT-PCR with GAPDH served as the internal control. (D) The culture conditioned media of TG2-silenced cells were collected and normalized by cell numbers prior to gelatin zymography analysis. (E) After TG2 knockdown, a wound healing assay was performed by scratching the cell layer with a pipette tip, and phase-contrast images were taken at 0 h and 24 h later to assess cell migration into the open space. Quantitative data are presented as the mean (± SD) percentage of migration distance (n = 20). * and # indicate a significant difference compared with the respective control (*p <*0.05).

To understand whether TG2 plays a role in the induction of the EMT process in A431 cells, we employed two experimental approaches. The first involved the transfection of TG2 siRNA into A431-P and A431-III cells. We found that knockdown of endogenous TG2 resulted in the reduced expression of various mesenchymal markers, namely fibronectin, vimentin, N-cadherin, and Snail (a key transcriptional repressor promoting EMT process). This knockdown had a greater effect on the A431-III subline than on A431-P cells as was shown by immunoblotting and confocal microscopy analysis (Figures 1A & 1B). In addition, and consistent with our previous study [26], knockdown of TG2 decreased the expression and activity of MMP-9, and this reduced the cells' migratory activity; these finding were obtained by RT-PCR, gelatin zymography and *in vitro *wound healing assays, respectively (Figures 1C to 1E).

**Figure 2:**
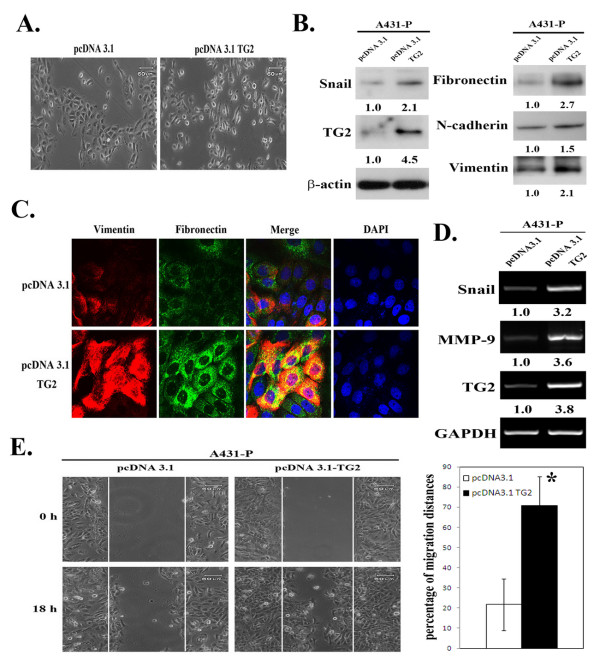
**Effect of TG2 over-expression on mesenchymal-like phenotype in A431-P cells**. (A) Phase-contrast images of empty vector (pcDNA3.1) or full length TG2 (pcDNA3.1-TG2)-transfected cells cultured on six-wells plates in culture medium containing 10% FBS (×100 magnification). At 48 h post-transfection, (B) cell lysates were prepared and subjected to immunoblotting analysis for TG2, Snail, fibronectin, N-cadherin, and vimentin. (C) Cells were immuno-stained for fibronectin (green) and vimentin (red) as well as having DAPI (blue) staining of the nuclei. The fluorescence images were visualized by confocal microscopy. (D) Total mRNA was extracted and analyzed for TG2, Snail and MMP-9 by RT-PCR. (E) Cell migratory activity was determined using the wound healing assay as described in figure 1. Quantitative data are presented as the mean (± SD) percentage of migration distance (n = 20). * indicates a significant difference compared with the control (*p <*0.05).

Next, we used the alternative approach of over-expressing TG2 in A431-P cells that show a naturally low level of TG2 (Figure 1A) by transfection with full-length TG2 (pcDNA3.1-TG2). A431-P cells normally produce compact clusters of cells in culture, and these clusters became more scattered and fibroblastic in nature following TG2 over-expression (Figure 2A). These changes were accompanied by increased expression of various mesenchymal markers, fibronectin, vimentin, N-cadherin and Snail (Figures 2B & 2C). Additionally, the A431-P cells over-expressing TG2 showed an increased expression of MMP-9 as well as displaying enhanced migratory potential (Figures 2D & 2E). Collectively, these results suggest that TG2 induces the acquisition of an EMT-like phenotype in A431-P and A431-III cells.

### Involvement of PI3K/Akt-GSK3 signaling in the TG2-facilitated EMT process

**Figure 3:**
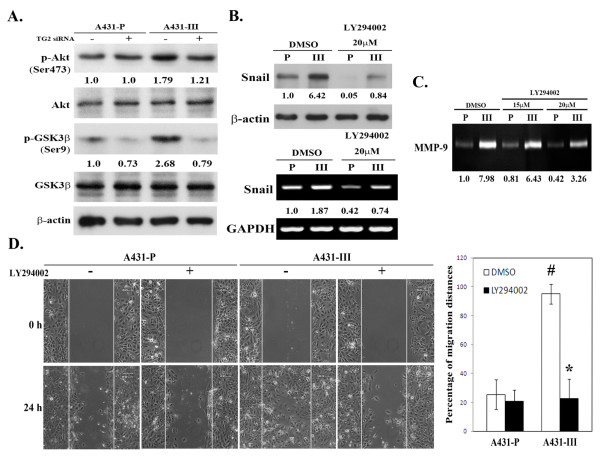
**Positive association of PI3K/Akt-GSK-3β signaling activation with the EMT phenotype in A431-P and A431-III cells**. (A) Cells were treated with 40 nM of control or specific TG2 siRNA. Cellular activity of Akt and **GSK-3β **were determined by analyzing their phosphorylation status using immunoblotting. (B-D) Cells were treated with 20 μM Akt inhibitor LY294002 for 24 h. (B) The cellular protein and RNA levels of Snail were respectively determined by immunoblotting and RT-PCR. (C) The secreted activity of MMP-9 was measured using gelatin zymography. (D) Cell migratory activity was determined by wound healing assay. Quantitative data are presented as the mean (± SD) percentage of migration distance (n = 20). * indicates a significant difference compared with the respective control (*p <*0.05). # indicates a significant difference compared with the A431-P (*p *< 0.05).

Recent studies have demonstrated that activation of PI3K/Akt-GSK-3β signaling may induce the EMT process, a loss of cell-to-cell adhesion and cell polarity, morphological changes, an induction of cell motility, and decreased cell-matrix adhesion [31]. GSK-3β, a ubiquitously expressed protein serine kinase, is active in resting epithelial cells [32], and inhibition of GSK-3β activity or its expression may lead to the EMT [33]. We therefore were interested to explore the role of PI3K/Akt- GSK-3β signaling in the TG2-facilitated EMT process in A431 cells. We first examined Akt and GSK-3β activity and their relationship with TG2. A431-III cells showed a relatively higher level of phosphorylated Akt-S473 (activation) and an increased level of phosphorylated GSK-3β-S9 (inactivation) when compared with A431-P cells (Figure 3A). In addition, knockdown of TG2 resulted in decreased Akt activity and increased GSK-3β activity in A431-III cells (Figure 3A).

**Figure 4:**
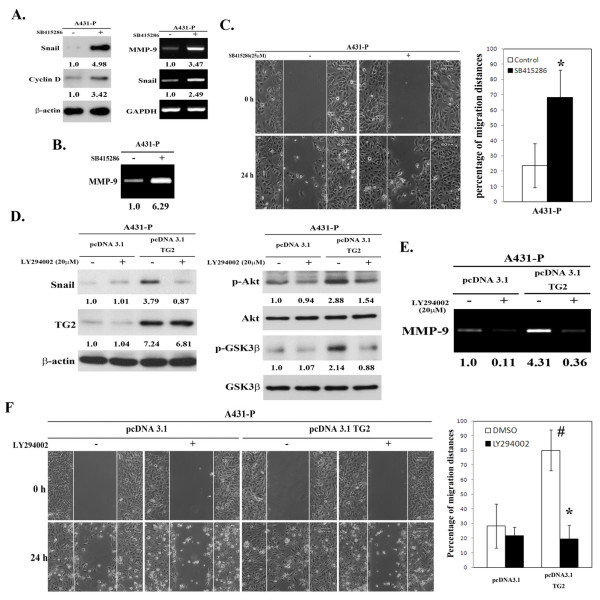
**Upregulation of PI3K/Akt-GSK-3β signaling activation is associated with the EMT phenotype in TG2-overexpressing A431-P cells**. (A-C) The cells were treated with 25 μM of the specific GSK3 inhibitor SB415286 for 48 h. (A) The cellular protein and RNA levels of Snail and MMP-9 were respectively determined by immunoblotting and RT-PCR. Cyclin D served as the indicator of the inhibition of GSK-3β activity. (B) The secreted MMP-9 activity was detected using gelatin zymography. (C) Cell migratory activity was determined by the wound healing assay. Quantitative data are presented as the mean (± SD) percentage of migration distance (n = 20). (D-F) A431-P cells were transfected with empty pcDNA3.1 vector or pcDNA3.1-TG2, and then treated with 20 μM of PI3K inhibitor LY294002 for 24 h. (D) Cell lysates were analyzed for phosphorylated Akt, GSK-3β, Snail and TG2 using immunoblotting. (E) The secreted activity of MMP-9 was detected by gelatin zymography. (F) Cell migratory activity was determined using the wound healing assay. Quantitative data are presented as the mean (± SD) percentage of migration distance (n = 20). * indicates a significant difference compared with the respective control (*p <*0.05). # indicates a significant difference compared with the A431-P (*p *< 0.05).

Next, we examined the potential involvement of PI3K/Akt-GSK3 signaling in the EMT using specific inhibitors of PI3K (LY294002) and GSK-3β (SB415286). Treatment of A431-III cells with LY294002 reduced the level of Snail and secreted MMP-9, and this was accompanied by reduced cell motility (Figures 3B to 3D). In parallel, treatment of A431-P cells with SB415286 increased the expression of Snail and MMP-9, as well as promoting cell motility (Figures 4A to 4C). Cyclin D was used as a positive control as it is subject to GSK-3β-dependent proteolysis [34]. To further ascertain the involvement PI3K/Akt-GSK-3β signaling in the TG2-induced acquisition of the mesenchymal phenotype, we used the alternative approach of transfecting pcDNA3.1-TG2 into A431-P cells. TG2-overexpresson in A431-P cells resulted in increased Akt activity and attenuated GSK-3β activity, and these effects were abrogated by treatment with the PI3K inhibitor LY294002 (Figures 4D). In a similar manner to that observed for A431-III cells, treatment of TG2-overexpressing A431-P cells with LY294002 reduced the level of Snail and secreted MMP-9, as well as reducing cell motility (Figures 4D to 4F). These results together suggest that the TG2 induced-acquisition of an EMT-like phenotype by the highly invasive A431-III subline involves an activation of PI3K/Akt signaling and an inactivation of GSK-3β.

### Involvement of NF-κB signaling in TG2-facilitated EMT process

**Figure 5:**
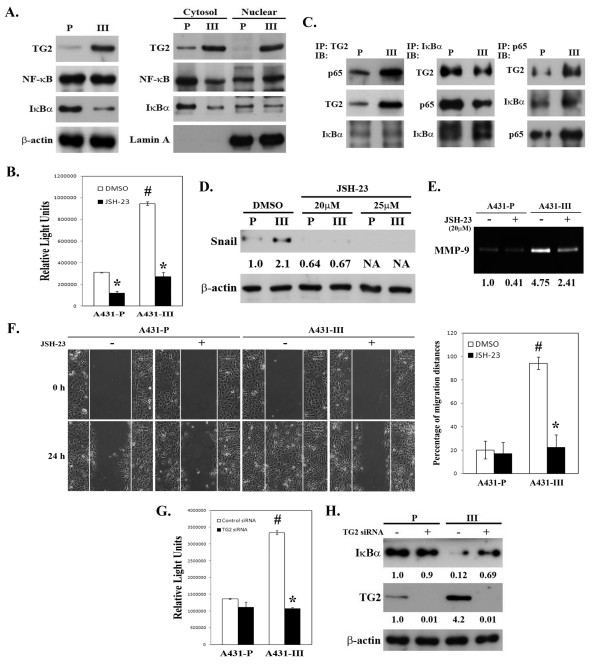
**Positive association of GSK-3β activity with TG2 and the EMT phenotype in A431-P and A431-III cells**. (A) Total cell lysates and cytosolic and nuclear extracts were prepared and analyzed for TG2, NF-κB, and IκBα by immunoblotting. (B) Cells were treated with 25 μM of JSH-23 for 24 h, and the cellular NF-κB of activity was determined using a luciferase reporter assay. (C) The interaction of TG2, NF-κB, and IκBα in A431-P and the A431-III sub-line. (D-E) Cells were treated with 20 or 25 μM of JSH-23 for 24 h, and cell lysates were analyzed for Snail by immunoblotting, and the conditioned media was analyzed for MMP-9 activity by gelatin zymography. (F) Cells were treated with 25 μM of JSH-23 for 24 h, and analyzed for migratory activity using wound healing assay. Quantitative data are presented as the mean (± SD) percentage of migration distance (n = 20). (G) Cells were transfected with control or specific TG2 siRNA, and cellular NF-κB activity was determined using a luciferase reporter assay. * indicates a significant difference compared with the respective control (*p *< 0.05). # indicates a significant difference compared with the A431-P (*p *< 0.05). (H) Cellular protein levels of IκBα and TG2 were detected by immunoblotting.

Wirth et al. identified NF-κB as a central mediator of the EMT in a mouse model of breast cancer progression [35]. In order to elucidate the role of NF-κB signaling in the TG2-facilitated EMT process in A431 cells, we conducted experiments using three approaches. The first was to examine NF-κB activity and its relationship with EMT. When compared to A431-P cells, the A431-III subline, which exhibits relatively high TG2 expression, showed a markedly reduced level of IκBα (an endogenous inhibitor of NF-κB), and an increased nuclear level of NF-κB relative to a similar total cellular level of NF-κB (Figure 5A). Using a NF-κB luciferase reporter assay, we found that NF-κB activity was significantly increased in A431-III cells, and this was suppressed by treatment with an NF-κB inhibitor, JSH-23 (Figure 5B). The increased NF-κB activity in A431-III cells was positively correlated with the increased nuclear level of TG2 (Figure 5A), and an increased association of TG2 with NFκB (Figure 5C). Additionally, treatment with JSH-23 reduced the level of Snail, secreted MMP-9 activity and the A431-III subline migratory activity (Figures 5D to 5F), which suggests the potential involvement of NF-κB in the EMT process.

**Figure 6:**
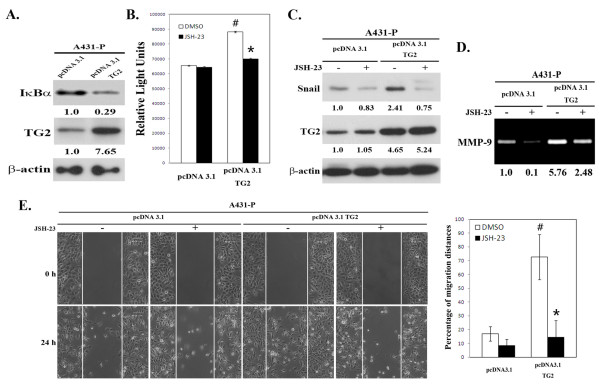
**Upregulation of NF-κB activity is associated with the EMT phenotype in TG2-overexpressed A431-P cells**. (A) A431-P cells were transfected with empty pcDNA3.1 vector or pcDNA3.1-TG2. Cellular protein levels of IκBα and TG2 were determined at 48 h post-transfection by immunoblotting. (B-E) Cells were treated with or without 25 μM of specific NF-κB inhibitor JSH-23 for 24 h. (B) Cellular NF-κB activity was determined using a luciferase reporter assay. (C) Cellular protein levels of Snail and TG2 were detected by immunoblotting. (D) The secreted activity of MMP-9 was analyzed by gelatin zymography. (E) Cell migratory activity was determined using a wound healing assay. Quantitative data are presented as the mean (± SD) percentage of migration distance (n = 20). * indicates a significant difference compared with the respective control (*p *< 0.05). # indicates a significant difference compared with the A431-P (*p *< 0.05).

Next, we explored the effect of TG2 siRNA transfection on A431-P and A431-III cells. Knockdown of TG2 led to a decrease of NF-κB activity and an increase in IκBα by the A431-III subline cells (Figures 5G & 5H), suggesting that upregulation of TG2 may induce NF-κB activity through a reduction of its inhibitor, IκBα. To further confirm this, we used an alternative approach whereby we transfected pcDNA3.1-TG2 into A431-P cells. TG2 over-expressing A431-P cells exhibited a depressed level of IκBα, which was accompanied by elevated NF-κB activity (Figures 6A & 6B). In a similar manner to A431-III cells, treatment of TG2-overexpressing A431-P cells with the NF-κB inhibitor JSH-23 reduced the level of Snail, secreted MMP-9 activity, and migratory activity (Figures 6C to 6E). These results together indicate that the TG2-induced acquisition of an EMT-like phenotype by the highly invasive A431-III cells involves the activation of NF-κB signaling.

## Discussion

Metastasis is a very complex and highly intriguing process. While some of the associated molecular mechanisms have begun to be unraveled, many remain to be understood. In order to further reveal some of the elusive factors that contribute to this complex process, our laboratory has established a model system using an A431 parental tumor cells (A431-P) and a highly invasive derivative subline (A431-III) [25].

To date, the role of TG2 in the EMT process is not well understood. Evidence from a limited number of studies has revealed that TG2 localized to the cell surface serves as a co-receptor for fibronectin by simultaneously associating with fibronectin and its receptor integrin, mainly β_1 _and β_3_, and that this process is independent of the catalytic activity of TG2 [36,37]. This process may in turn promote cell adhesion, spread, and migration [36,38]. We have further documented the increased expression of TG2 and Snail in the A431-III tumor subline that are both associated with the acquisition of the mesenchymal-like phenotype [26]. Additionally, the upregulated expression of TG2 in A431-III cells not only enhances the association of fibronectin with β integrin, but also induces the expression of fibronectin [26]. Moreover, we presented an interesting hypothesis whereby Snail and MMP-9 form a mutual positive regulatory loop in highly invasive A431-III cells that may account for the facilitation of the EMT progression [27]. The present study further shows that TG2 seems to be able to positively regulate the expression of Snail in A431 cells (Figures 1 & 2), which suggests that TG2 may act, at least in part, through an upregulation of Snail in order to promote the EMT process. The molecular mechanism of TG2 involvement in the acquisition of the mesenchymal-like phenotype in A431-III cells still awaits further investigation.

Previous studies have documented that the aberrant expression of TG2 in epithelial cells results in constitutive activation of focal adhesion kinase (FAK), Akt, and NF-κB [13,19,20,39]. These pathways are involved in the regulation of the EMT, in conferring drug resistance, and in promoting metastasis [35,40,41]. The aberrant activation of Akt signaling is widely implicated in many human cancers. Several studies have reported that activation of the PI3K/Akt-GSK-3β signaling pathway is also a central feature of the EMT, and that this involves accumulation of Snail in the nucleus [33]. As compared to its parental cells, the highly invasive A431-III subline displays increased Akt activity together with a concomitant reduction in GSK-3β activity, as well as having increased Snail and MMP-9 levels (Figure 3). Inhibition of PI3K activity (LY294002) in A431 tumor cells attenuated the TG2-induced activation of Akt and GSK-3β, and suppressed the increases in Snail, MMP-9, and cell motility (Figure 4). Additionally, inhibition of GSK-3 activity (SB415286) in A431-P cells elevated the expression of Snail and MMP9, and enhanced cell motility (Figure 4). These results support the concept that TG2 acts in part through an activation of PI3K/Akt signaling in A431 tumor cells that inhibits GSK-3β, and therefore upregulates Snail/MMP-9 and ultimately promotes EMT progression. Verma et al. documented that overexpression of TG2 in cancer cells is associated with a constitutive activation of focal adhesion kinase (FAK) and its downstream PI3K/Akt pathway [16]. Our earlier study also showed that the highly invasive A431-III subline exhibits increased FAK activity compared to its parental cells [25], and that knockdown of endogenous TG2 reduced the phosphorylation activation of FAK-Tyr397 (data not shown). Collectively, these results imply that TG2 may sequentially activate FAK and PI3K/Akt- in A431 tumor cells, which in turn induces the acquisition of a mesenchymal phenotype by A431 tumor cells.

Constitutive activation of NF-κB is known to confer resistance to cell death-inducing stimuli, including chemotherapeutic agents [42], and to promote metastasis by inducing EMT [35]. As compared to its parental cells, the highly invasive A431-III subline has increased NF-κB activity that is largely due to a reduction of IκBα, and this has been positively associated with TG2 expression (Figures 5 & 6). In addition to the increase in TG2 level in the A431-III subline, there was also an increased association of TG2 with p65 NF-κB (Figure 5). These results agree with the findings of other researchers who have shown that drug-resistant cancer cells have increased levels of TG2, which enhances NF-κB activity, in turn, through a novel mechanism, namely the formation of a TG2- NF-κB complex [13,15,20,43]. In this study, we also demonstrated that there was an inhibition NF-κB activity (JSH-23) in A431 tumor cells, which led to a suppression of the TG2-induced increases in Snail, MMP-9, and cell motility (Figures 5 & 6). We proposed that TG2 acts at least in part through an activation of NF-κB to upregulate Snail and MMP-9; this then boosts the acquisition of mesenchymal characteristics by the A431 tumor cells.

It is believed that cancer cells are at intermediate states in the EMT process. Indeed, once cells have invaded the primary tumor and penetrated the surrounding tissue, they must be able to colonize at a new tissue site. To achieve this, cancer cells may undergo a mesenchymal-epithelial transition (MET) resulting in a reformation of the epithelial phenotype in terms of cell-cell adhesion [44,45]. A cancer cell that attains plasticity and also shifts through the EMT and MET may account for many of the difficulties associated with cancer clinical therapy.

## Conclusions

In a previous study, we described a rapid method to probe, explore and learn about the EMT. As enunciated elsewhere, we obtained a highly invasive A431-III sub-line from A431-P cells via Boyden chamber selection. Both A431-P and A431-III cells exerted EMT characteristics, while A431-III cells have acquired a mesenchymal-like phenotype, and also exhibit an elevated expression of Snail, one of the prime factors involved in the acquisition of the mesenchymal-like phenotype [27]. In the present study, we have demonstrated that the expression of Snail is associated with enhanced NF-κB and PI3K/Akt-GSK-3β and that TG2 participates in the acquisition of a mesenchymal transition. We conjecture that the enhancement of Snail expression by TG2 induces the acquisition in A431-III cells of a mesenchymal-like phenotype that then promotes the secretion of MMP-9, which enhances cancer cell motility and increases metastatic potential. These observations also support our contention that that TG2 is a promising therapeutic target for reversing drug resistance and inhibiting the early advent of metastasis of tumor cells.

## List of Abbreviations

TG2: Tissue Transglutaminase; MMP-9: Matrix Metalloproteinase-9; EMT: Epithelial-Mesenchymal Transition; GSK-3: Glycogen Synthase Kinase-3.

## Competing interests

The authors declare that they have no competing interests.

## Authors' contributions

Conceived and designed the experiments: CYL and MTL. Performed the experiments: CYL and PHT Data analyzed: GDC, CHC, CJH, PPL and CYL. Paper writing and editing: CYL, JJH and CCK. All authors have read and approved the final manuscript.

## References

[B1] Thiery JP Epithelial-mesenchymal transitions in tumour progression Nat Rev Cancer 2002 10 442 454 10.1038/nrc822 12189386

[B2] Thiery JP Epithelial-mesenchymal transitions in development and pathologies Curr Opin Cell Biol 2003 10 740 746 10.1016/j.ceb.2003.10.006 14644200

[B3] Yilmaz M Christofori G EMT, the cytoskeleton, and cancer cell invasion Cancer Metastasis Rev 2009 10 15 33 10.1007/s10555-008-9169-0 19169796

[B4] Iwatsuki M Mimori K Yokobori T Ishi H Beppu T Nakamori S Baba H Mori M Epithelial-mesenchymal transition in cancer development and its clinical significance Cancer Sci 2010 10 293 299 10.1111/j.1349-7006.2009.01419.x 19961486 PMC11159985

[B5] Huber MA Kraut N Beug H Molecular requirements for epithelial-mesenchymal transition during tumor progression Curr Opin Cell Biol 2005 10 548 558 10.1016/j.ceb.2005.08.001 16098727

[B6] Guarino M Epithelial-mesenchymal transition and tumour invasion Int J Biochem Cell Biol 2007 10 2153 2160 10.1016/j.biocel.2007.07.011 17825600

[B7] Peinado H Portillo F Cano A Transcriptional regulation of cadherins during development and carcinogenesis Int J Dev Biol 2004 10 365 375 10.1387/ijdb.041794hp 15349812

[B8] Fesus L Szondy Z Transglutaminase 2 in the balance of cell death and survival FEBS Lett 2005 10 3297 3302 10.1016/j.febslet.2005.03.063 15943974

[B9] Fesus L Piacentini M Transglutaminase 2: an enigmatic enzyme with diverse functions Trends Biochem Sci 2002 10 534 539 10.1016/S0968-0004(02)02182-5 12368090

[B10] Lorand L Graham RM Transglutaminases: crosslinking enzymes with pleiotropic functions Nat Rev Mol Cell Biol 2003 10 140 156 10.1038/nrm1014 12563291

[B11] Collighan RJ Griffin M Transglutaminase 2 cross-linking of matrix proteins: biological significance and medical applications Amino Acids 2009 10 659 670 10.1007/s00726-008-0190-y 18982407

[B12] Ientile R Caccamo D Griffin M Tissue transglutaminase and the stress response Amino Acids 2007 10 385 394 10.1007/s00726-007-0517-0 17390097

[B13] Verma A Mehta K Transglutaminase-mediated activation of nuclear transcription factor-kappaB in cancer cells: a new therapeutic opportunity Curr Cancer Drug Targets 2007 10 559 565 10.2174/156800907781662275 17896921

[B14] Mehta K High levels of transglutaminase expression in doxorubicin-resistant human breast carcinoma cells Int J Cancer 1994 10 400 406 10.1002/ijc.2910580316 7914183

[B15] Verma A Mehta K Tissue transglutaminase-mediated chemoresistance in cancer cells Drug Resist Updat 2007 10 144 151 10.1016/j.drup.2007.06.002 17662645

[B16] Verma A Wang H Manavathi B Fok JY Mann AP Kumar R Mehta K Increased expression of tissue transglutaminase in pancreatic ductal adenocarcinoma and its implications in drug resistance and metastasis Cancer Res 2006 10 10525 10533 10.1158/0008-5472.CAN-06-2387 17079475

[B17] Cao L Petrusca DN Satpathy M Nakshatri H Petrache I Matei D Tissue transglutaminase protects epithelial ovarian cancer cells from cisplatin-induced apoptosis by promoting cell survival signaling Carcinogenesis 2008 10 1893 1900 10.1093/carcin/bgn158 18667446 PMC2556973

[B18] Hwang JY Mangala LS Fok JY Lin YG Merritt WM Spannuth WA Nick AM Fiterman DJ Vivas-Mejia PE Deavers MT Clinical and biological significance of tissue transglutaminase in ovarian carcinoma Cancer Res 2008 10 5849 5858 10.1158/0008-5472.CAN-07-6130 18632639 PMC2547344

[B19] Herman JF Mangala LS Mehta K Implications of increased tissue transglutaminase (TG2) expression in drug-resistant breast cancer (MCF-7) cells Oncogene 2006 10 3049 3058 10.1038/sj.onc.1209324 16449978

[B20] Kim DS Park SS Nam BH Kim IH Kim SY Reversal of drug resistance in breast cancer cells by transglutaminase 2 inhibition and nuclear factor-kappaB inactivation Cancer Res 2006 10 10936 10943 10.1158/0008-5472.CAN-06-1521 17108131

[B21] Verma A Guha S Diagaradjane P Kunnumakkara AB Sanguino AM Lopez-Berestein G Sood AK Aggarwal BB Krishnan S Gelovani JG Mehta K Therapeutic significance of elevated tissue transglutaminase expression in pancreatic cancer Clin Cancer Res 2008 10 2476 2483 10.1158/1078-0432.CCR-07-4529 18413840

[B22] Han JA Park SC Reduction of transglutaminase 2 expression is associated with an induction of drug sensitivity in the PC-14 human lung cancer cell line J Cancer Res Clin Oncol 1999 10 89 95 10.1007/s004320050247 10190315 PMC12199886

[B23] Shao M Cao L Shen C Satpathy M Chelladurai B Bigsby RM Nakshatri H Matei D Epithelial-to-mesenchymal transition and ovarian tumor progression induced by tissue transglutaminase Cancer Res 2009 10 9192 9201 10.1158/0008-5472.CAN-09-1257 19951993

[B24] Kumar A Xu J Brady S Gao H Yu D Reuben J Mehta K Tissue transglutaminase promotes drug resistance and invasion by inducing mesenchymal transition in mammary epithelial cells PLoS One 2010 10 e13390 10.1371/journal.pone.0013390 20967228 PMC2953521

[B25] Kao WT Lin CY Lee LT Lee PP Hung CC Lin YS Chen SH Ke FC Hwang JJ Lee MT Investigation of MMP-2 and -9 in a highly invasive A431 tumor cell sub-line selected from a Boyden chamber assay Anticancer Res 2008 10 2109 2120 18751383

[B26] Chen SH Lin CY Lee LT Chang GD Lee PP Hung CC Kao WT Tsai PH Schally AV Hwang JJ Lee MT Up-regulation of fibronectin and tissue transglutaminase promotes cell invasion involving increased association with integrin and MMP expression in A431 cells Anticancer Res 2010 10 4177 4186 21036738

[B27] Lin CY Tsai PH Kandaswami CC Lee PP Huang CJ Hwang JJ Lee MT Matrix metalloproteinase-9 cooperates with transcription factor Snail to induce epithelial-mesenchymal transitioncancer science 2011 10 815 827 10.1111/j.1349-7006.2011.01861.x 21219539

[B28] Giard DJ Aaronson SA Todaro GJ Arnstein P Kersey JH Dosik H Parks WP In vitro cultivation of human tumors: establishment of cell lines derived from a series of solid tumors Journal of the National Cancer Institute 1973 10 1417 1423 4357758 10.1093/jnci/51.5.1417

[B29] Bradford MM A rapid and sensitive method for the quantitation of microgram quantities of protein utilizing the principle of protein-dye binding Anal Biochem 1976 10 248 254 10.1016/0003-2697(76)90527-3 942051

[B30] Schreiber E Matthias P Muller MM Schaffner W Rapid detection of octamer binding proteins with 'mini-extracts', prepared from a small number of cells Nucleic Acids Res 1989 10 6419 10.1093/nar/17.15.6419 2771659 PMC318318

[B31] Grille SJ Bellacosa A Upson J Klein-Szanto AJ van Roy F Lee-Kwon W Donowitz M Tsichlis PN Larue L The protein kinase Akt induces epithelial mesenchymal transition and promotes enhanced motility and invasiveness of squamous cell carcinoma lines Cancer Res 2003 10 2172 2178 12727836

[B32] Papkoff J Aikawa M WNT-1 and HGF regulate GSK3 beta activity and beta-catenin signaling in mammary epithelial cells Biochem Biophys Res Commun 1998 10 851 858 10.1006/bbrc.1998.8888 9647782

[B33] Bachelder RE Yoon SO Franci C de Herreros AG Mercurio AM Glycogen synthase kinase-3 is an endogenous inhibitor of Snail transcription: implications for the epithelial-mesenchymal transition J Cell Biol 2005 10 29 33 15631989 10.1083/jcb.200409067 PMC2171685

[B34] Diehl JA Cheng M Roussel MF Sherr CJ Glycogen synthase kinase-3beta regulates cyclin D1 proteolysis and subcellular localization Genes Dev 1998 10 3499 3511 10.1101/gad.12.22.3499 9832503 PMC317244

[B35] Huber MA Azoitei N Baumann B Grunert S Sommer A Pehamberger H Kraut N Beug H Wirth T NF-kappaB is essential for epithelial-mesenchymal transition and metastasis in a model of breast cancer progression J Clin Invest 2004 10 569 581 15314694 10.1172/JCI21358 PMC503772

[B36] Akimov SS Belkin AM Cell surface tissue transglutaminase is involved in adhesion and migration of monocytic cells on fibronectin Blood 2001 10 1567 1576 10.1182/blood.V98.5.1567 11520809

[B37] Chhabra A Verma A Mehta K Tissue transglutaminase promotes or suppresses tumors depending on cell context Anticancer Res 2009 10 1909 1919 19528447

[B38] Akimov SS Krylov D Fleischman LF Belkin AM Tissue transglutaminase is an integrin-binding adhesion coreceptor for fibronectin J Cell Biol 2000 10 825 838 10.1083/jcb.148.4.825 10684262 PMC2169362

[B39] Verma A Guha S Wang H Fok JY Koul D Abbruzzese J Mehta K Tissue transglutaminase regulates focal adhesion kinase/AKT activation by modulating PTEN expression in pancreatic cancer cells Clin Cancer Res 2008 10 1997 2005 10.1158/1078-0432.CCR-07-1533 18381937

[B40] Thiery JP Acloque H Huang RY Nieto MA Epithelial-mesenchymal transitions in development and disease Cell 2009 10 871 890 10.1016/j.cell.2009.11.007 19945376

[B41] Kalluri R Weinberg RA The basics of epithelial-mesenchymal transition J Clin Invest 2009 10 1420 1428 10.1172/JCI39104 19487818 PMC2689101

[B42] Orlowski RZ Baldwin AS Jr NF-kappaB as a therapeutic target in cancer Trends Mol Med 2002 10 385 389 10.1016/S1471-4914(02)02375-4 12127724

[B43] Mann AP Verma A Sethi G Manavathi B Wang H Fok JY Kunnumakkara AB Kumar R Aggarwal BB Mehta K Overexpression of tissue transglutaminase leads to constitutive activation of nuclear factor-kappaB in cancer cells: delineation of a novel pathway Cancer Res 2006 10 8788 8795 10.1158/0008-5472.CAN-06-1457 16951195

[B44] Peinado H Olmeda D Cano A Snail, Zeb and bHLH factors in tumour progression: an alliance against the epithelial phenotype? Nat Rev Cancer 2007 10 415 428 10.1038/nrc2131 17508028

[B45] Hugo H Ackland ML Blick T Lawrence MG Clements JA Williams ED Thompson EW Epithelial--mesenchymal and mesenchymal--epithelial transitions in carcinoma progression J Cell Physiol 2007 10 374 383 10.1002/jcp.21223 17680632

